# Changes of cerebral cortical structure and cognitive dysfunction in “healthy hemisphere” after stroke: a study about cortical complexity and sulcus patterns in bilateral ischemic adult moyamoya disease

**DOI:** 10.1186/s12868-021-00672-x

**Published:** 2021-11-14

**Authors:** Ziqi Liu, Shihao He, Yanchang Wei, Ran Duan, Cai Zhang, Tian Li, Ning Ma, Xin Lou, Rong Wang, Xiaoyuan Liu

**Affiliations:** 1 Department of Neurosurgery, Beijing Tiantan Hospital, Capital Medical University, Beijing, 100070 China; 2 Center of Stroke, Beijing Institute for Brain Disorders, Beijing, 10069 China; 3 Department of Neurosurgery, Peking University International Hospital, Beijing, 102206 China; 4 Collaborative Innovation Center of Assessment for Basic Education Quality, Beijing Normal University, Beijing, 100875 China; 5 Department of Radiology, Chinese PLA General Hospital, Beijing, China

**Keywords:** Moyamoya disease, Stroke, SBM, Cortical complexity, Fractal dimension, Cognitive dysfunction

## Abstract

**Background:**

Moyamoya disease (MMD) is an uncommon cerebrovascular disease which leads to progressive stenosis and occlusion of the bilateral internal carotid artery and main intracerebral arteries. Concerns are always on how the hemisphere with infarction affects cognitive function, while little attention is paid to the role that the non-infarcted hemisphere plays. Therefore, we aimed to detect cortical indexes, especially cortical complexity in the left or right hemisphere separately in patients with MMD after stroke.

**Methods:**

28 patients with MMD (14 males, 14 females) and 14 healthy controls were included in this study. All participants underwent cognitive tests and magnetic resonance imaging (MRI) scan. The preprocessing of three-dimensional T1 weighted images were performed by standard surface-based morphometry. Surface-based morphometry statistical analysis was carried out with a threshold of False Discovery Rate (FDR) *P* < 0.05 and fractal dimension (FD) was used to provide a quantitative description of cerebral cortical complexity.

**Results:**

Widespread cognitive dysfunctions were found in MMD patient with stroke. Extensive FD reduction in the left hemisphere with right-sided infarction, mainly in the superior temporal, inferior frontal, and insula, while the post central gyrus, superior parietal, and inferior parietal gyrus also showed a wide range of significant differences (FDR corrected P < 0.05). Meanwhile, FD changes in the right hemisphere with left-sided infarction are restricted to the precuneus and cingulate isthmus (FDR corrected P < 0.05).

**Conclusions:**

Extensive cognitive impairment was reconfirmed in Moyamoya disease with stroke, while wild and asymmetrical decrease of cortical complexity is observed on both sides. These differences could be relative to unbalanced cognitive dysfunction, and may be the result of a long-term chronic ischemia and compensatory of the contralateral hemisphere to the infarction.

**Supplementary Information:**

The online version contains supplementary material available at 10.1186/s12868-021-00672-x.

## Background

Moyamoya disease (MMD) is an uncommon cerebrovascular disease which leads to progressive stenosis and occlusion of the bilateral internal carotid artery, middle cerebral artery, anterior artery and even posterior artery [1]. The outcome is often severe cerebrovascular accidents, especially cerebral infarction. The mechanism of physical function injury and recovery in patients with cerebral infarction has been widely studied. So, more and more researchers begin to focus on relatively undetectable cognitive dysfunction. Previous studies have found that the proportion of cognitive injury caused by cerebral infarction is as high as 30–50% [2, 3], and cognitive recovery of post-stroke within 6 month is less optimistic [4]. Cognitive impairment can have a significant impact on quality of life and daily life activities by reducing the degree of independence of individuals [5], and is associated with long-term occupancy and disability, so it should be more thoroughly studied. Concerns are always on how the hemisphere with infarctions affects cognitive function, while little attention is paid to the role that the non-infarcted hemisphere plays.

In previous studies, both gray matter and white matter were found to be secondary impaired to varying degrees in patients with MMD without stroke [6–8], and different types of cognitive dysfunction are relative to cortical or subcortical impairments [9]. However, it was interesting to see if the relatively “healthy hemispheres” of patients with infarction also had similar changes. Therefore, we aimed to detect the cortical indexes such as gyrification index (GI), cortical complexity, cortical thickness and sulcus depth in the relatively normal left or right hemisphere separately in patients with MMD after stroke with surface-based morphometry (SBM), by means of neuroimaging, especially volumetric T1 in Magnetic Resonance Imaging (MRI), which is a non-invasive method to study brain structure.

Brain structure analysis generally used voxel-based morphometry (VBM) to investigate changes in gray matter (GM) [10]. However, this well-established method needs a whole brain structural analysis that examines local changes in gray matter volume (GMV), not for left or right hemisphere respectively. Freesurfer [11] is another well-known toolbox to progress and analysis structural MRI data for its high accuracy and separation, while it cost too much time (about 6–8 h) for each subject. Therefore, we seek another toolbox named Computational Anatomy Toolbox 12 (CAT12) [12] with both advantages of VBM and Freesurfer. There is evidence [13] suggesting that although cortical thickness estimations were systematically higher in CAT12, CAT12 delivers accurate cortical estimates and can be considered a fast and reliable alternative to FreeSurfer. Meanwhile, unlike FreeSurfer, CAT12 can reconstruct the central surface directly by using projection-based thickness (PBT) method [14]. Numbers of studies [15–17] have been carried out with CAT12 and the results were fairly reliable.

The concept of fractal dimension (FD) has been largely used to describe the geometric properties of complex objects made up of parts that are similar to the whole in some ways [18]. Fractal dimension was thought to be a great sensitivity to detect cortical atrophy and age-related effects [19, 20], and provided distinct information from traditional indices such as cortical thickness and gyrification in all kinds of psychiatric diseases [21–26].Cortical complexity can be considered a measure of gyrification by combining multifactorial messages from folding frequency, sulcal depth, convolution of gyral shape, and cortical thickness into a single value [27, 28]. It reflects properties such as dendrite complexity and synaptic density, which are highly correlated with cognitive ability.

We hypothesized that cerebral infarction in unilateral hemisphere resulted in significant cognitive impairment and that cortical surface index changes could be found in the non-infarcted hemisphere. These changes may be asymmetrical and associated with the type or location of cognitive impairments. Cortical complexity may be one of the most characteristic changes, reflecting long-term cerebral ischemia and cognitive compensation on the non-infarcted side.

## Results

### Cognitive test

In the left hemisphere with right -sided infarction, compared with the control group, there was a statistical difference in the mental rotation (ROT), while there were significant statistical differences in Raven's Standard Progressive Matrices (RSPM), verbal working memory 2 (VWM2), simple subtraction (SUB), complex subtraction (COMSUB) and word-memory (WORDM). Meanwhile, in the right hemisphere with left-sided infarction, compared with the control group, there were statistical differences in RSPM, SUB, WORDM, and PICTM, whereas there were significant statistical differences in ROT, COMSUB, VWM1, and VWM2. See Table 1 for details.

**Table 1:** Cognitive results of patients and controls

Variables	Patients with	Patients with	Controls	Statistics	
	right stroke(*n* = 14)	left stroke(*n* = 14)	(*n* = 14)	*P values*	
	Mean ± SD	Mean ± SD	Mean ± SD	right stroke	left stroke
CRT_RT	525.07 ± 60.926	539.57 ± 150.79	473.86 ± 138.776	0.222	0.241
CRT_ACC	94.71 ± 10.823	97.50 ± 3.956	99.14 ± 1.460	0.152	0.164
RSPM	13.79 ± 6.852	16.00 ± 6.288	30.79 ± 21.491	0.009**	0.02*
ROT	14.29 ± 8.579	11.57 ± 9.796	23.07 ± 10.866	0.025*	0.007**
VWM1	7.14 ± 1.994	6.07 ± 2.235	8.21 ± 1.369	0.109	0.005**
VWM2	4.21 ± 2.045	4.64 ± 1.781	7.79 ± 1.424	0**	0**
SUB	27.14 ± 14.464	29.86 ± 13.341	41.57 ± 9.196	0.004**	0.012*
COMSUB	11.36 ± 7.571	11.00 ± 6.504	25.57 ± 10.338	0**	0**
WORDM	52.64 ± 16.463	57 ± 19.275	70.50 ± 7.552	0.002**	0.026*
PICTM	69.71 ± 14.626	70.43 ± 8.187	76.43 ± 2.243	0.112	0.018*
EXCUT1	0.07 ± 3.430	0.36 ± 2.061	− 0.29 ± 2.431	0.753	0.457
EXCUT2	− 2.29 ± 3.931	− 1.57 ± 3.106	3.07 ± 2.586	0.538	0.177

*SD* standard deviation, *CRT_RT/ACC* Choice reaction time_ reaction time/ accuracy, *RSPM* Raven's Standard Progressive Matrices, *ROT* Mental rotation, *VWM* verbal working memory, digit span, 1, Recite in order, 2, Recite in reverse order, *SUB* Simple subtraction, *COMSUB* Complex subtraction, *WORDM* word-memory, *PICTM* picture-memory, *EXCUT* Executive function,1, same direction, 2, Opposite direction; Keep only three decimals, and display as 0 if less than 0.001; *P < 0.05, **P < *0.01*

### SBM analysis

**Fig. 1:**
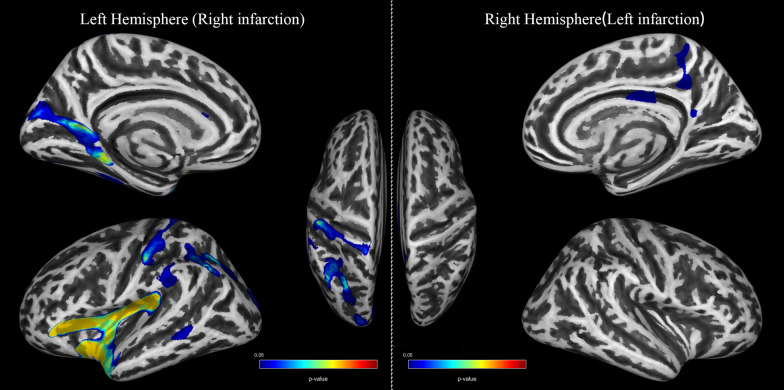
Cortical areas of decreased cortical complexity in MMD patients (TFCE, FDR corrected *P* < *0.05*). Left hemisphere stands for the unilateral left hemisphere of patients with right infarction and Right hemisphere stands for the unilateral right hemisphere of patients with right infarction

On the one hand, MMD demonstrated extensive FD reduction in the left hemisphere with right -sided infarction, mainly in the superior temporal, inferior frontal, and insula, while the post central gyrus, superior parietal, and inferior parietal gyrus also showed a wide range of significant differences (TFCE, FDR corrected *P* < *0.05*). On the other hand, changes in the right hemisphere with left-sided infarction are restricted to the precuneus and cingulate isthmus (TFCE, FDR corrected *P* < *0.05*). No matter left or right infarction, significant reductions in FD were observed in the contralateral precuneus, post central gyrus, and cingulate isthmus. No positive results are found in correlation analyses between Suzuki stages and FD. See Tables 2, 3 and Fig. 1 for more details.

**Table 2:** Participants information

Variables	Patients with	Patients with	Controls	Statistics
	right stroke(*n* = 14)	left stroke(*n* = 14)	(*n* = 14)	*P values*
	Mean ± SD	Mean ± SD	Mean ± SD	
Sex (M: F)	7:7	7:7	7:7	1
Age (year)	44.14 ± 12.50	38.36 ± 7.55	39.14 ± 11.22	0.306
Education(year)	9.5 ± 2.47	10.79 ± 3.12	10.86 ± 3.98	0.468
Medical history, n (%)				
Hypertension	2(14%)	2(14%)	4(29%)	0.539
Coronary heart disease	0	1(7%)	2(14%)	0.341
Diabetes	1(7%)	1(7%)	1(7%)	1
Dyslipidemia	2(14%)	1(7%)	3(21%)	0.558
Smoking history	2(14%)	3(21%)	3(21%)	0.857
Alcohol taking	2(14%)	1(7%)	3(21%)	0.558
Suzuki Stage				
1	1	0		
2	4	2		
3	4	8		
4	5	2		
5	0	2		
6	0	0		

Values are numbers of cases (%) unless otherwise indicated. Mean values are presented with SDs

*F* female, *M* male, *SD* standard deviation

**Table 3:** Cortical areas of decreased cortical complexity in MMD patients with right or left infarction compared to controls

*P-value*	Size(vertices)	Overlap of atlas region*	
Left hemisphere (Right infarction)			
0.02418	10,064	40%	Insula
		16%	Superior temporal
		10%	Pars triangularis
		9%	Supramarginal
		7%	Lateral orbitofrontal
		6%	Temporal pole
		5%	Postcentral
		2%	Transverse temporal
		2%	Pars opercularis
0.02418	3432	25%	Cuneus
		19%	Precuneus
		18%	Lingual
		17%	Isthmus cingulate
		16%	Parahippocampal
		4%	Pericalcarine
0.02733	3079	50%	Inferior parietal
		40%	Superior parietal
		10%	Supramarginal
0.02562	2638	100%	Postcentral
0.04408	860	100%	supramarginal
0.04142	720	83%	Fusiform
		18%	Inferior temporal
0.03743	648	83%	Lateral occipital
		17%	Superior parietal
0.04469	410	60%	Middle temporal
		40%	Bankssts
Right hemisphere (Left infarction)			
0.04067	868	82%	Precuneus
		9%	Isthmus cingulate
		9%	Paracentral
0.04067	378	100%	Posterior cingulate

Decreased cortical complexity in MMD with right or left infarction (*TFCE, FDR-corrected P* < *0.05*)

^*^Only show regions size more than 100 vertices (Size × Overlap)

**Fig. 2:**
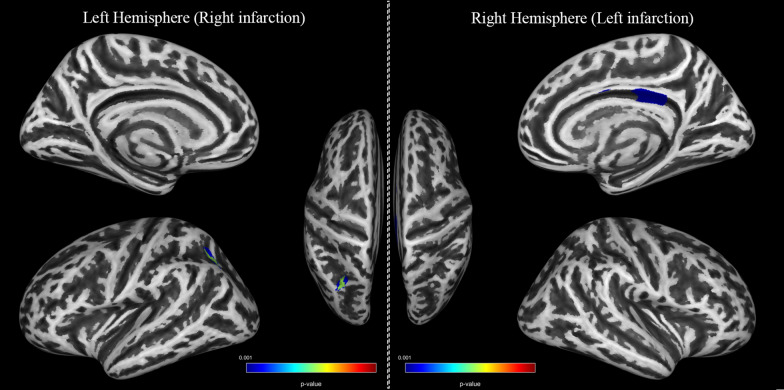
Cortical areas of decreased sulcus depth in MMD patients (TFCE, uncorrected *P* < *0.001*). Left hemisphere stands for the unilateral left hemisphere of patients with right infarction and Right hemisphere stands for the unilateral right hemisphere of patients with right infarction

Conversely, significant differences in gyrification, sulcus depth and cortical thickness were not observed in the relatively healthy hemispheres (TFCE, FDR corrected *P* < *0.05*). However, using a less rigorous statistical threshold (TFCE, uncorrected *P* < *0.001*), decreased sulcus depth areas with significant differences in posterior cingulate, isthmus cingulate in the right with left-sided infarction and superior parietal, inferior parietal in the left with right-sided infarction can be observed. No positive results are observed in correlation analyses between Suzuki stages and sulcus depth. More information in detail could be found in Fig. 2.

## Discussion

Our study is the first one using morphological analysis to evaluate the gray matter microstructure rather than volume in the contralateral hemisphere to the infarction separately in adult MMD. In precious study, just two related articles showed morphological changes in adult MMD. Through FSL (FSL-VBM, version 4.1, http://www.fmrib.ox.ac.uk/fsl), Ken Kazumata [8] has found decreased gray matter density in the bilateral posterior cingulate cortex, but lack of surface indexes analysis and explanation. Next, decreased volume of corpus callosum, bilateral subcortical nuclei (thalamus, putamen, caudate), and hippocampus are observed following revascularization surgery using Freesurfer (version 5.1.0; surfer.nmr.mgh.harvard.edu) [9]. However, only volume and cortical thickness are estimated. Apart from these two studies, no more attention was paid to brain structure recently especially gray matter in MMD. Therefore, we refer some other mental diseases and neuropathies for our discussion.

Not surprisingly, many cognitive impairments were found in patients with MMD due to cerebral infarction on one side of the brain, while morphological changes in the contralateral side of non-infarction also existed. Cortical changes in the left hemisphere appear to be more widespread than in the right. This led us to wonder if the difference between dominant and non-dominant hemispheres can fully explain the phenomenon. In the following parts, we will discuss each indicator with significant differences in detail.

Deeply analyzing the results of cognitive function, verbal working memory and picture-memory deficits are greater in patients with left infarction than the right side, while simple subtraction, intelligence and word-memory are similarly worse in patients with right infarction than the left side. For the difference of left and right lesions, we suspect that it is related to whether the lesions touch the dominant hemisphere of a certain cognitive function. Previous studies have reported that patients with left hemisphere cerebral infarction have a worse prognosis [29–31]. In a cognitive study involving nine sub-tests [32], cognitive impairment and sleep quality impairment caused by middle cerebral artery stroke were observed in the left and right hemispheres, regardless of gender. In another previous study investigating motor observation and imagination ability of hemispheric stroke patients [33], patients with left subcortical lesions recruited more cortical regions when processing motor images and videos, suggesting that lesions in the dominant hemisphere had a greater impact on the motor system. However, another recent studies [34, 35] showed that visuospatial working memory deficits were greater in patients with right hemisphere infarction than in patients with left hemisphere infarction. Similarly, studies [36] have shown that imaginative psychology, such as mental rotation, tends to be biased towards the right hemisphere. These studies all prove that there is asymmetry in cognitive impairment, and the asymmetry of different cognitive functions may be an important reason for the distinguish in the degree of impairment.

The decrease of FD reflects lower cortical complexity, which may be related to the unreduced cortical thickness and cognitive impairment. In a study of FD value of human cortical surface [27], researchers found a negative correlation between cortical FD value and cortical thickness. The frequency of folds and convolution of gyral shape will increase, rather than deepen sulcal regions when the FD is increased in cerebral cortex. This is consistent with previous findings [37] that cortical cellular growth would occur preferentially along tangential axes which is the path of least resistance. These previous findings are according with our result. Cortical complexity has also been proved to be correlated with cognitive function and age [38]. The complexity of adult cerebral cortex is lower than that of adolescents [39], and the complex shape of cortical surface is significantly related to intelligence and education level. In our study, there were more reasons to suspect that reduction of cortical complexity could be caused by long-term ischemic states of bilateral hemispheres due to chronic stenosis or occlusion of intracarotid artery and/or middle brain artery which is characteristic of MMD, while more evidences of blood flow combined with cognitive function are needed to prove our hypothesis.

## Limitations

Our study also had some limitations. Firstly, the small sample limited the wildly use of our results, and a larger number of samples were needed to support our conclusions. Secondly, we studied bilateral ischemic Moyamoya disease with stroke, and the effect of stroke on cognition has been clearly demonstrated. However, further study of the contribution to cognitive impairment of the relatively healthy cerebral hemisphere is required. Third, due to the different size and location of cerebral infarction, quantitative measurement cannot be carried out, so we can only roughly remove the injured side of the cerebral hemisphere in our study. These patients have yet to be studied using more advancing methods to investigate the mechanisms of cognitive impairment. Finally, restricted to the small sample size, correlation analyses between vascular stages and SBM parameters do not provide a strong power. Larger sample sizes are needed to prove the relationship and presumption in discussion part.

## Conclusions

Extensive cognitive impairment was reconfirmed in Moyamoya disease with stroke, while wild FD decrease, on behalf of cortical complexity, was asymmetrical on both sides. These differences could cause unbalanced cognitive dysfunction in unilateral hemisphere separately, and may be the result of a long-term chronic ischemia and compensatory of the contralateral hemisphere to the infarction.

## Method

### Participants

This prospective study was approved by the research ethics committee of Beijing Tiantan Hospital affiliated to Capital Medical University (KYSQ2019-058-01). Written informed consent was obtained from all participants. The study included 28 patients with MMD (14 males, 14 females) from the Neurosurgery Department of Beijing Tiantan Hospital which is affiliated to the Capital Medical University and Peking University International Hospital between October 2018 and December 2019. Moreover, the control group included 14 volunteers (7 males, 7 females). There was no significant difference in age, education level, sex composition, and risk factors between patient group and control group (*P* > *0.10*). More details of statistical result could be found in Table 2.

The inclusion criteria of patients were as follows: (1) All patients should follow the Guidelines for Diagnosis and Treatment of Moyamoya Disease (Spontaneous Occlusion of the Circle of Willis), the research committee on the pathology and treatment of spontaneous occlusion of the circle of Willis; [40] (2) In the MMD patients group, there must be previous ischemic or hemorrhagic attack in only either left or right hemisphere more than 3 months but less than 6 months ago, but no new ischemic or hemorrhagic attack within 3 months. The lesion area should be more than 1.5 cm; (3) Righthand dominance; (4) Being free of dementia, or depression; and (5) no major psychiatric disease or other medical conditions.

The exclusion criteria of patients were as follows: (1) Acute stage of cerebral infarction within 3 month or any infarction happened in both hemispheres, other neuropsychiatric diseases and severe systemic diseases (e.g., AD, Parkinson’s disease); (2) Any contraindications for MR scans (e.g., metal implants); (3) Manifestation of any medications that could affect cognitive abilities; (4) Fatigue or hunger; (5) An inability to complete tasks independently.

By means of social recruitment, we released recruitment advertisements for the control group. By asking medical history, we recorded clinical variables such as age, sex and past medical history, and conducted cognitive test after MRI examination. 14 people were finally included in the control group and matched with the MMD group.

Inclusion criteria for the control group were as follows: no clinical evidence of psychiatric or neurological disease, no brain damage on routine MRI, and no history of drugs use that could affect cognitive function.

### MRI parameters

MRI data were obtained using a 3.0-Tesla MR system (Verio A Tim + Dot System, Siemens, Germany). Volumetric T1 (three-dimension, 3D) gradient echo was acquired with a thickness of 1.0 mm (Voxel size = 1.0 × 1.0 × 1.0 mm, flip angle = 8°, time of repetition (TR) = 2300 ms, time of echo (TE) = 3.25 ms, matrix = 256 × 256, FOV = 250 × 250 mm, FOV phase = 100%). T2-weighted images were acquired with a thickness of 8 mm (flip angle = 150 degrees, TR = 5000 ms, TE = 98 ms, FOV = 220 × 220 mm).

### Neuropsychological assessments

For the neurocognitive function test, we adopted the method of Liu et al. [7]. All cognitive assessment programs were tested using the Online Psychological Experimental System. The participants were tested using computer workstations by only one neuropsychologist who was blinded to the clinical data. The interval time between the neuropsychological test and MRI examination was less than 3 days. More specific details about the neurocognitive function test could be found in the Additional file 1.

### Surface-based morphometry analysis

The structural MRI data preprocessing was performed in a standard manner by Computational Anatomy Toolbox 12 (CAT12, http://www.neuro.unijena.de/cat/,version r1450) in statistical parametric mapping 12 (SPM12, http://www.fil.ion.ucl.ac.uk/spm/software/spm12/, version 7219) using MATLAB 2014b software (MathWorks, Natick, Massachusetts, USA). The CAT12 toolbox for SPM contains a series of fully automated pipelines for processing surface-based morphometry that allows the measurement of cortical thickness and reconstruction of the central surface in one step. Secondly, before and after progressing, all images are checked though “Check data quality” in CAT12 in order to ensure the image quality and homogeneity. Then, central surface parameters, such as gyrification index(GI) [41], FD [42] and sulcus depth, are then extracted or calculated for both hemispheres respectively which is described in Yotter [42] and Louders [41]. The GI and FD were computed following the manual established by Gaser and Kurth (http://www.neuro.unijena.de/cat12/CAT12-Manual.pdf). At last, all surface measures for both hemispheres were merged and resampled to a higher resolution mesh (164 k) that is compatible with Freesurfer data. Resample and Smooth with a Gaussian kernel of 15 mm are performed for all surface-based parameters for both hemispheres separately, prior to the second-level analyses.

### Statistical analysis

The analyses of clinical characteristics were conducted using ANOVA for age, education and a χ^2^ test for sex ratio and medical history. Two-sample t-test was used for cognitive results for left and right hemispheres groups separately. All above were analyzed in SPSS 19.0 (SPSS Inc., Chicago, Illinois, USA).

To determine differences between groups, a two-sample t-test was performed through the batch-mode implemented in SPM12, adjusting for total intracranial volume (TIV) as covariates of no interest. Contrasts were processed using 5000 times displacement test after Threshold-Free Cluster Enhancement (TFCE) [43] and statistical significance was defined as *P* < 0.05 FDR (False Discovery Rate) corrected for cortical complexity or uncorrected *P* < 0.001 for gyrification index, cortical thickness and sulcus depth. All significant results were presented on Desikan-Killiany Atlas (DKA) template for left or right hemisphere respectively [44].

## Supplementary Information

**Additional file 1.** Neuropsychological assessments.

## Data Availability

The datasets used and analyzed during the current study are available from the corresponding author on reasonable request.

## Abbreviations

MMD: Moyamoya disease
SBM: Surface-based morphometry
MRI: Magnetic Resonance Imaging
VBM: Voxel-based morphometry
GMV: Gray matter volume
TFCE: Threshold-Free Cluster Enhancement
WM: White matter
PBT: Projection-based thickness
SPM: Statistical Parametric Mapping software
DKA: Desikan-Killiany Atlas
SD: Standard deviation
CRT_RT/ACC: Choice reaction time_ reaction time/ accuracy
RSPM: Raven's Standard Progressive Matrices
ROT: Mental rotation
VWM: Verbal working memory
SUB: Simple subtraction
COMSUB: Complex subtraction
WORDM: Word-memory
PICTM: Picture-memory
EXCUT: Executive function
VBM: Voxel-based morphometry
MRI: Magnetic Resonance Imaging
CAT12: Computational Anatomy Toolbox 12
GI: Gyrification index
FD: Fractal dimension
CT: Cortical thickness
FDR: False Discovery Rate
PD: Parkinson disease
