# Supplementary material for: Changes of cerebral cortical structure and cognitive dysfunction in “healthy hemisphere” after stroke: a study about cortical complexity and sulcus patterns in bilateral ischemic adult moyamoya disease
Source: BMC Neurosci. 2021 Nov 14;22:66. doi: 10.1186/s12868-021-00672-x (PMC8590755; doi:10.1186/s12868-021-00672-x)
Supplement: Supplementary file 1 — Additional file 1. Neuropsychological assessments. [file 12868_2021_672_MOESM1_ESM.docx]

# Neuropsychological assessments

Choice reaction time (RT) was used as the baseline condition and the index of movement ability. The objective of using the basic RT task was to determine manual response effect and main processing speed. The choice RT task was adapted from the simple RT task from Butterworth’s Dyscalculia Screener (2003). [1] In all 30 trials of this task, a white fixation cross and a white dot were presented on a black screen. The former was presented in the center of the screen, and the latter was presented on the left or right side of the fixation cross (It was present on the left side in half the cases). Participants should press “Q” or “P” with their left or right fingers if the dot appeared on the left or right side of the fixation cross, respectively. The interstimulus interval was randomized between 1500 and 3000 ms. Nonverbal matrix reasoning was used to assess general intelligence and reasoning ability. The task was adapted from the abstract reasoning ability part of Raven’s Progressive Matrices (Raven, 2000). The mental rotation was used to evaluate the visual-spatial ability [2]. Verbal working memory was used to measure working memory capacity [3]. A multistep, multilocation search task was used to evaluate executive function [4]. Simple and complex subtraction problems were used to assess simple calculation ability and magnitude representation [5]. Word-memory ability and visual short-term memory were measured with the short-term memory (STM) span for Chinese words and phrases and the picture STM test, respectively [6]. The Edinburgh Handedness Inventory was used to investigate left and right-handedness [7]. The AD8 questionnaire was used to determine the degree of cognitive decline in daily life [8]. Additional details on the cognitive assessment questions can be found elsewhere [9].

References：

1. Butterworth B. Dyscalculia screener.London: NFER Nelson Publishing Company Ltd. 2003.

2. STEVEN G. VANDENBERG' ARK. MENTAL ROTATIONS, A GROUP TEST OF THREE-DIMENSIONAL SPATIAL VISUALIZATION. Perceptual and Motor Skills. 1978;47:599-604.

3. Heather M. Conklin BS, Clayton E. Curtis, Joanna Katsanis, and William G. Iacono. Verbal Working Memory Impairment in Schizophrenia Patients and Their First-Degree Relatives: Evidence From the Digit Span Task. Am J Psychiatry. 2000;157:275-7.

4. Zelazo PD, Reznick JS, Spinazzola J. Representational flexibility and response control in a multistep multilocation search task. Developmental Psychology. 1998;34(2):203-14.

5. Zhou X, Wei W, Zhang Y, Cui J, Chen C. Visual perception can account for the close relation between numerosity processing and computational fluency. Front Psychol. 2015;6:1364.

6. Yu B, Simon HA. STM SPAN FOR CHINESE WORDS AND PHRASES. Acta Psychologica Sinica. 1985.

7. Dellatolas G, De Agostini M, Curt F, Kremin H, Letierce A, Maccario J, et al. Manual skill, hand skill asymmetry, and cognitive performances in young children. Laterality. 2003;8(4):317-38.

8. J.E. Galvin MCMRKKPMAC, RN, MSN; S.J. Muich, RN, MSN; E. Grant; J.P. Miller; M. Storandt; and J.C. Morris. The AD8 A brief informant interview to detect dementia. NEUROLOGY. 2005;65:559-64.

9. Cui J, Zhang Y, Wan S, Chen C, Zeng J, Zhou X. Visual form perception is fundamental for both reading comprehension and arithmetic computation. Cognition. 2019;189:141-54.
